# Performance of Different Versions of Duke Criteria in Diagnosing Infective Endocarditis in Patients With Intracardiac Prosthetic Materials

**DOI:** 10.1093/ofid/ofaf507

**Published:** 2025-08-19

**Authors:** Matthaios Papadimitriou-Olivgeris, Bruno Ledergerber, Jana Epprecht, Berit Siedentop, Pierre Monney, Michelle Frank, Georgios Tzimas, Nicolas Fourré, Virgile Zimmermann, Giulia Domenichini, Lars Niclauss, Matthias Kirsch, Mathias Van Hemelrijck, Omer Dzemali, Benoit Guery, Barbara Hasse

**Affiliations:** Infectious Diseases Service, Lausanne University Hospital and University of Lausanne, Lausanne, Switzerland; Infectious Diseases Service, Hospital of Valais and Institut Central des Hôpitaux, Sion, Switzerland; Department of Infectious Diseases and Hospital Epidemiology, University Hospital Zurich and University of Zurich, Zurich, Switzerland; Department of Infectious Diseases and Hospital Epidemiology, University Hospital Zurich and University of Zurich, Zurich, Switzerland; Department of Infectious Diseases and Hospital Epidemiology, University Hospital Zurich and University of Zurich, Zurich, Switzerland; Department of Cardiology, Lausanne University Hospital and University of Lausanne, Lausanne, Switzerland; Department of Cardiology, University Hospital Zurich and University of Zurich, Zurich, Switzerland; Department of Cardiology, Lausanne University Hospital and University of Lausanne, Lausanne, Switzerland; Infectious Diseases Service, Lausanne University Hospital and University of Lausanne, Lausanne, Switzerland; Infectious Diseases Service, Lausanne University Hospital and University of Lausanne, Lausanne, Switzerland; Department of Cardiology, Lausanne University Hospital and University of Lausanne, Lausanne, Switzerland; Department of Cardiac Surgery, Lausanne University Hospital and University of Lausanne, Lausanne, Switzerland; Department of Cardiac Surgery, Lausanne University Hospital and University of Lausanne, Lausanne, Switzerland; Department of Cardiac Surgery, University Hospital Zurich and University of Zurich, Zurich, Switzerland; Department of Cardiac Surgery, University Hospital Zurich and University of Zurich, Zurich, Switzerland; Department of Cardiac Surgery, City Hospital of Zurich - Triemli, Zurich, Switzerland; Center for Experimental and Translational Cardiology, University of Zurich, Zurich, Switzerland; Infectious Diseases Service, Lausanne University Hospital and University of Lausanne, Lausanne, Switzerland; Department of Infectious Diseases and Hospital Epidemiology, University Hospital Zurich and University of Zurich, Zurich, Switzerland

**Keywords:** Duke criteria, European society of cardiology (ESC), infective endocarditis, international society for cardiovascular infectious diseases (ISCVID), prosthetic valve

## Abstract

**Background:**

Diagnosing infective endocarditis (IE) is a significant challenge. This study aimed to compare the diagnostic performance of the 2015 and 2023 European Society of Cardiology (ESC) and the 2023 International Society for Cardiovascular Infectious Diseases (ISCVID) Duke clinical criteria in a cohort of patients with suspected IE and intracardiac prosthetic material.

**Methods:**

This retrospective study was conducted at 2 Swiss University Hospitals (2014–2024). The reference standard was the diagnosis of the Endocarditis Team or expert clinicians. Patients with IE (reference standard) classified as definite IE by the Duke criteria were considered true positives, while those without IE classified as rejected IE were considered true negatives.

**Results:**

Of the 1025 episodes with suspected IE and intracardiac prosthetic material, 537 (61%) had IE. Using the 2015 ESC, 2023 ESC, and 2023 ISCVID clinical criteria, 324 (32%), 367 (36%), and 430 (42%) episodes were classified as definite IE, respectively. The sensitivity for the 2015 Duke-ESC, 2023 Duke-ESC, and 2023 Duke-ISCVID the clinical criteria was calculated to be 56% (95% CI: 52–60%), 61% (57–65%), and 71% (67–74%), respectively, while the specificity 67% (63–71%), 56% (51–61%), and 34% (29–38%), respectively.

**Conclusions:**

The 2023 ISCVID Duke clinical criteria demonstrated the highest sensitivity for diagnosing IE compared to the 2015 and 2023 ESC Duke criteria in patients with intracardiac prosthetic material. However, this was at the expense of specificity.

## Teaser

Among 1046 episodes of suspected infective endocarditis, the sensitivity of 2023 Duke-ESC and 2023 Duke-ISCVID clinical criteria was higher compared to 2015 Duke-ESC criteria; the specificity of the 2 2023 versions was lower compared to 2015 Duke-ESC.

The epidemiology of infective endocarditis (IE) has shifted over recent decades, largely due to the increasing use of prosthetic valves and cardiac implantable electronic devices (CIEDs) [1–3]. More recently, transcatheter aortic valve implantation (TAVI) has gained prominence, becoming the preferred treatment for elderly patients who are ineligible for surgery or at high to intermediate surgical risk [4, 5].

Diagnosing IE in patients with prosthetic intracardiac material remains challenging despite advancements in microbiology and imaging techniques. For many years, transesophageal echocardiography (TEE) was the cornerstone of diagnosing prosthetic valve endocarditis (PVE). However, its sensitivity in detecting PVE is lower than for native valve endocarditis, particularly in identifying paravalvular abscesses. In patients with CIED, differentiating true vegetations from fibrin or thrombotic deposits on leads by TOE further complicates the diagnosis [6, 7].

The introduction of newer imaging modalities, such as [^18^F]Fluorodeoxyglucose Positron Emission Tomography/Computed Tomography ([^18^F]FDG PET/CT) and cardiac CT, has improved IE detection, particularly in patients with prosthetic intracardiac material [8, 9]. When combined with TEE, these techniques enhance the identification of paravalvular complications, which are more frequent in PVE [10, 11]. However, cardiac CT has no role in diagnosing CIED-related IE, and [^18^F]FDG PET/CT has low sensitivity for detecting lead involvement [12].

These imaging advancements have been incorporated into the evolving Duke criteria. The 2015 European Society of Cardiology (ESC) modifications introduced positive cardiac CT (for native or prosthetic valves) and positive [^18^F]FDG PET/CT (for prosthetic valves, if performed at least 3 months after implantation) as major imaging criteria [13]. In 2023, the International Society for Cardiovascular Infectious Diseases (ISCVID) and ESC provided distinct versions by expanding the applicability of [^18^F]FDG PET/CT to native valves and CIED-related IE and adding TAVI and CIED in the minor predisposing criterion [14, 15]. The 2023 ISCVID version further refined the criteria by adding a minor imaging criterion for [^18^F]FDG PET/CT positivity within 3 months of prosthetic valve implantation, while the 2023 ESC criteria classified a positive [^18^F]FDG PET/CT as a major imaging criterion, even if performed within 3 months postimplantation [14, 15]. Lastly, 2023 ISCVID made important modifications in the major microbiological criterion, including several pathogens as typical microorganisms only in the presence of intracardiac prosthetic material, such as coagulase-negative staphylococci [14].

One of the key challenges in assessing the diagnostic accuracy of the Duke diagnostic lies in the “possible IE” category, cases that remain indeterminate, often do not prompt immediate treatment, but still warrant further diagnostic workup. Most published studies have taken a conservative approach by classifying all episodes labeled as “possible IE” by the Duke criteria, but lacking a confirmed clinical diagnosis of IE, as true negatives [16–23]. This approach assumes that indeterminate findings, in the absence of confirmatory evidence, should not compromise the criteria's specificity. However, a previous study on enterococcal bacteremia proposed an alternative strategy: reclassifying such “possible IE” cases without a final diagnosis of IE as false positives [24]. This method more accurately reflects the inherent diagnostic uncertainty and highlights the clinical need for additional evaluation before deciding whether these cases warrant management as true IE.

Despite these refinements, few studies have evaluated the performance of different Duke criteria versions in patients with prosthetic intracardiac material [18, 19, 21, 25, 26]. Most existing studies are limited by small sample sizes and an overrepresentation of IE cases, leading to an overestimation of diagnostic sensitivity. This study aims to assess the diagnostic accuracy of the 2015 and 2023 Duke clinical criteria in patients with suspected IE and prosthetic intracardiac material.

## METHODS

This multicenter study performed in 2 Swiss hospitals, Lausanne University Hospital (CHUV) and University Hospital Zurich (USZ), was based on merging 3 cohorts:

- CHUV's retrospective cohort of patients with bacteremia/candidemia from January 2015 to December 2021.
- CHUV's cohort of patients with suspected IE, spanning from January 2014 to June 2024, with retrospective inclusion of IE patients from January 2014 to December 2017 and prospective inclusion of patients with suspected IE from January 2018 to June 2024.
- USZ's cohort of patients with possible/proven IE from USZ, spanning from January 2014 to June 2024, with retrospective inclusion from January 2014 to December 2017 and prospective inclusion from January 2018 to June 2024.

Inclusion criteria were adult patients with suspected IE (episodes with blood cultures drawn and echocardiography performed specifically to diagnose IE), absence of data refusal for the retrospective cohort, and presence of written consent for the prospective one. Exclusion criterion was the absence of prosthetic intracardiac material. Prosthetic intracardiac material was defined as surgical prosthetic valve, TAVI, CIED, and LVAD. Patients with a surgical prosthetic valve or TAVI were considered to have sufficient cardiac imaging if they underwent [¹⁸F]FDG PET/CT or cardiac CT and had cardiac surgery, or, in the absence of these, if echocardiography showed evidence of vegetation, perforation, abscess, aneurysm, pseudoaneurysm, or fistula. Patients without any of these echocardiographic findings were also considered to have sufficient imaging if the risk of IE was deemed low by the infectious diseases consultant responsible for the case.

We collected demographic, clinical, imaging, and microbiological information from patients' health records. Since January 2018, in both centers, IE diagnosis was based on the evaluation of each center's Endocarditis Team. Prior to 2018, each case underwent classification as IE or not, based on the assessment of 4 expert clinicians (CHUV: M.P.-O., P.M.; USZ: M.V.H., B.H.). Cases were categorized as rejected, possible, or definite IE, following the 2015 ESC [13], 2023 ISCVID [14], and 2023 ESC [15] clinical criteria.

We used SPSS version 26.0 (SPSS, Chicago, IL, USA) for the statistical analyses. Mann–Whitney *U* test was used to assess continuous variables, while the χ² or Fisher exact test for examining categorical variables. Two different analyses were conducted to evaluate the performance of the Duke clinical criteria for diagnosing IE, using the diagnosis established by the Endocarditis Teams or expert clinicians as the reference standard. In the first analysis, patients with IE (per the reference standard) who were classified as having definite IE according to the Duke criteria were considered true positives, whereas those classified as possible or rejected IE were considered false negatives. Conversely, patients without IE (per the reference standard) who were classified as rejected or possible IE were considered true negatives, while those classified as definite IE were considered false positives. In the second analysis, the only difference concerned patients without IE (per the reference standard) who were categorized as possible IE by the Duke criteria: these were now considered false positives instead of true negatives. We calculated sensitivity, specificity, positive and negative predictive values (PPV, NPV), and accuracy with the corresponding 95% confidence interval (CI) in the whole cohort. All tests were 2-tailed, and *P* < .05 was considered statistically significant.

## RESULTS

**Figure 1.**
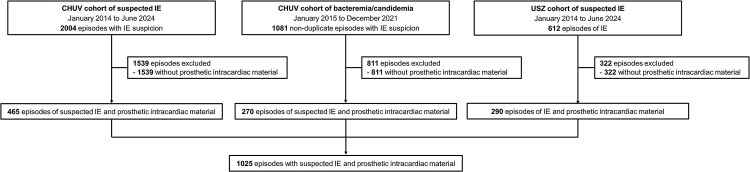
Flowchart of included patients from the 3 cohorts. CHUV, Lausanne University Hospital; USZ, University Hospital Zurich.

Among the 3697 episodes from the 3 cohorts, 1025 were included, consisting of 465 from the CHUV cohort of suspected IE, 270 non-duplicate episodes from the CHUV bacteremia/candidemia cohort, and 290 from the USZ IE cohort (Figure 1). The intracardiac prosthetic material was surgical prosthetic valve in 537 (52%) episodes, TAVI in 101 (10%), CIED in 566 (55%), and LVAD in 45 (4%). Thereof, 578 (56%) ultimately received a final IE diagnosis by each institution's Endocarditis Team or by the expert clinicians, with 355 (61%) being linked to prosthetic valves, 196 (34%) to CIED leads, and 112 (19%) to native valves; no case of infection related to LVAD cannulas was identified (Supplementary Table 1).

Transthoracic echocardiography (TTE), TEE, [^18^F]FDG PET/CT, and cardiac CT were performed in 956 (93%), 549 (54%), 306 (30%), and 53 (5%) episodes, respectively, with 336 episodes having [^18^F]FDG PET/CT and/or cardiac CT (Supplementary Table 1). Among the 620 episodes with surgical prosthetic valve or TAVI, 502 (81%) were considered to have sufficient cardiac imaging. Of these, 355 (71%) ultimately received a final diagnosis of IE, as determined by each institution's Endocarditis Team or expert clinicians. Among the 118 episodes without adequate cardiac imaging, the reasons were advanced age or comorbidities in 95 cases, patient refusal in 18, and death prior to imaging in 5 cases.

Table 1 shows the comparison of episodes with or without IE diagnosis among 1025 episodes with suspected IE and intracardiac prosthetic material. Table 2 presents patient characteristics stratified by the 3 versions of the Duke criteria. Using the 2015 ESC, 2023 ESC, and 2023 ISCVID clinical criteria, 324 (32%), 367 (36%), and 430 (42%) episodes were classified as definite IE, respectively. The 2015 ESC clinical criteria classified 354 (35%) episodes as possible IE, the 2023 ESC clinical criteria 381 (37%), and the 2023 ISCVID clinical criteria 432 (42%). Supplementary Table 2 depicts cases classified as IE by the Endocarditis Teams or expert clinicians and treated accordingly, despite being classified as rejected IE by any version of the Duke clinical criteria. Supplementary Table 3 presents cases classified by the Endocarditis Teams or IE expert clinicians as not having IE, despite being classified as definite IE by any Duke version.

**Table 1.** Comparison of Episodes With or Without Infective Endocarditis Diagnosis Among 1025 Episodes With Suspected Infective Endocarditis and Intracardiac Prosthetic Material

	No Infective Endocarditis (*n* = 447)	Infective Endocarditis (*n* = 578)	*P* Value
Demographics			
Male sex, *n* (%)	346 (77)	445 (77)	.881
Age (median y, IQR)	75 (67–82)	69 (55–78)	<.001
Cardiac predisposing factors			
Intravenous drug use, *n* (%)	7 (2)	30 (5)	.002
Rheumatic heart disease/hypertrophic cardiomyopathy, *n* (%)	1 (0.2)	5 (0.9)	.240
Congenital disease, *n* (%)	13 (3)	103 (18)	<.001
Surgical prosthetic valve, *n* (%)	180 (40)	357 (62)	<.001
Prior endocarditis, *n* (%)	46 (10)	126 (22)	<.001
Cardiac implantable electronic devices, *n* (%)	278 (62)	288 (50)	<.001
Transcatheter aortic valve implantation, *n* (%)	37 (8)	64 (11)	.141
Moderate or severe valve regurgitation/stenosis, *n* (%)	39 (9)	74 (13)	.044
Heart transplantation, *n* (%)	5 (1)	3 (0.5)	.306
Left ventricular assist device, *n* (%)	31 (7)	14 (2)	.001
Microbiological data			
Bacteremia/fungemia, *n* (%)	338 (76)	478 (83)	.006
*S. aureus*, *n* (%)	96 (22)	208 (36)	<.001
Coagulase-negative staphylococci, *n* (%)	22 (5)	41 (7)	.189
*Streptococcus* spp.	64 (14)	95 (16)	.385
*S. agalactiae*, *S. dysgalactiae*, *n* (%)	20 (5)	12 (2)	.031
*S. gallolyticus* or viridans streptococci, *n* (%)	39 (9)	81 (14)	.011
*Enterococcus* spp., *n* (%)	84 (19)	86 (15)	.108
Community-acquired enterococci without known primary focus, *n* (%)	10 (2)	69 (12)	<.001
*E. faecalis*, *n* (%)	52 (12)	72 (13)	.701
Gram-positive (other than staphylococci, streptococci, and enterococci), *n* (%)	12 (3)	19 (3)	.714
HACEK, *n* (%)	2 (0.4)	12 (2)	.029
Gram-negative (other than HACEK), *n* (%)	80 (18)	19 (3)	<.001
Fungi, *n* (%)	18 (4)	9 (2)	.018
Microorganisms that occasionally or rarely cause IE isolated from 3 or more separate blood culture sets, *n* (%)	25 (6)	66 (11)	.001
New typical microorganism in the presence of intracardiac prosthetic material, *n* (%)	81 (18)	71 (12)	.010
Positive culture from sterile site for typical pathogen, *n* (%)	20 (5)	20 (4)	.420
*Coxiella burnetii* antiphase I IgG titer ≥ 1:800, *n* (%)	0 (0)	5 (0.9)	.072
*Bartonella henselae/quintana* IgG titer ≥ 1:800, *n* (%)	1 (0.2)	1 (0.2)	1.000
Imaging data			
Positive echocardiography (either TTE or TEE) for vegetation, perforation, abscess, aneurysm, pseudoaneurysm, fistula, *n* (%)	9 (2)	372 (65)	<.001
Abnormal metabolic activity in [^18^F]FDG PET/CT in prosthetic valve, *n* (%)	3 (0.7)	92 (16)	<.001
Abnormal metabolic activity in [^18^F]FDG PET/CT in CIED lead, *n* (%)	0 (0)	16 (3)	<.001
Abnormal metabolic activity in [^18^F]FDG PET/CT in native valve, *n* (%)	0 (0)	10 (2)	.006
Positive cardiac-CT for vegetation, perforation, abscess, aneurysm, pseudoaneurysm, fistula, *n* (%)	1 (0.2)	29 (5)	<.001
Significant new valvular regurgitation on echocardiography as compared to previous imaging, *n* (%)	21 (5)	75 (13)	<.001
Leaflet thickening on echocardiography (either TTE or TEE) or cardiac CT, *n* (%)	12 (3)	59 (10)	<.001
Manifestations			
Fever, *n* (%)	328 (73)	445 (77)	.189
Immunological phenomena, *n* (%)	2 (0.4)	58 (10)	<.001
Vascular phenomena (major arterial emboli, septic pulmonary infarcts, mycotic aneurysm, intracranial hemorrhage, conjunctival hemorrhages, and Janeway's lesions), *n* (%)	24 (5)	224 (38)	<.001
Cerebral abscess, n (%)	0 (0)	5 (0.9)	.072
Hematogenous osteoarticular septic complications (spondylodiscitis, septic arthritis), *n* (%)	11 (3)	36 (6)	.004
Spondylodiscitis, *n* (%)	5 (1)	19 (3)	.023
Septic arthritis, *n* (%)	6 (1)	20 (4)	.043
Data on surgery/CIED-extraction/histopathology			
Valve surgery performed, *n* (%)	9 (2)	169 (92)	<.001
CIED-extraction (among 566 patients with CIED), *n* (%)	32 (12)	123 (43)	<.001
Autopsy performed, *n* (%)	5 (1)	13 (4)	.015
Histopathology compatible for IE, *n* (%)	0 (0)	63 (11)	<.001
Positive culture of vegetation, abscess, *n* (%)	0 (0)	118 (20)	<.001
Positive nucleic acid-based tests, *n* (%)	0 (0)	22 (4)	<.001
Macroscopic evidence of IE by inspection (surgery/autopsy), *n* (%)	0 (0)	105 (0)	<.001

Abbreviations: [^18^F]FDG PET/CT, [^18^F]Fluorodeoxyglucose Positron Emission Tomography/Computed Tomography; ESC, European Society of Cardiology; CIED, cardiac implantable electronic devices; HACEK, *Haemophilus* spp., *Aggregatibacter* spp., *Cardiobacterium hominis*, *Eikenella corrodens*, *Kingella kingae*; IE, infective endocarditis; IQR, interquartile range; TEE, transesophageal echocardiography; TTE, transthoracic echocardiography.

**Table 2.** Classifications Based on the 3 Versions of the Duke Clinical Criteria Among 1025 Episodes With Suspected Infective Endocarditis and Intracardiac Prosthetic Material

	No Infective Endocarditis (*n* = 447)	Infective Endocarditis (*n* = 578)
Duke major clinical criteria		
Microbiological (2015 ESC), *n* (%)	98 (22)	357 (62)
Microbiological (2023 ESC), *n* (%)	122 (27)	359 (62)
Microbiological (2023 ISCVID), *n* (%)	208 (47)	454 (79)
Imaging (2015 ESC), *n* (%)	10 (2)	430 (74)
Imaging (2023 ESC), *n* (%)	22 (5)	449 (78)
Imaging (2023 ISCVID), *n* (%)	29 (7)	453 (78)
Surgery (2023 ISCVID), *n* (%)	0 (0)	2 (0.3)
Duke minor clinical criteria		
Microbiological (2015 ESC), *n* (%)	114 (26)	28 (5)
Microbiological (2023 ESC), *n* (%)	58 (13)	15 (3)
Microbiological (2023 ISCVID), *n* (%)	89 (20)	20 (4)
Predisposition (2015 ESC), *n* (%)	195 (44)	395 (68)
Predisposition (2023 ESC), *n* (%)	439 (98)	576 (100)
Predisposition (2023 ISCVID), *n* (%)	447 (100)	578 (100)
Vascular (2015 ESC), *n* (%)	22 (5)	235 (41)
Vascular (2023 ESC), *n* (%)	30 (7)	249 (43)
Vascular (2023 ISCVID), *n* (%)	22 (5)	236 (41)
Immunological (all versions), *n* (%)	2 (0.4)	57 (10)
Fever (all versions), *n* (%)	328 (73)	445 (77)
Classification according to 2015 Duke-ESC clinical criteria		
Rejected, *n* (%)	300 (67)	47 (8)
Possible, *n* (%)	146 (33)	208 (36)
Definite, *n* (%)	1 (0.2)	323 (56)
Classification according to 2023 Duke-ESC clinical criteria		
Rejected, *n* (%)	250 (56)	27 (5)
Possible, *n* (%)	182 (41)	199 (34)
Definite, *n* (%)	15 (3)	352 (61)
Classification according to 2023 Duke-ISCVID clinical criteria		
Rejected, *n* (%)	151 (34)	12 (2)
Possible, *n* (%)	274 (61)	158 (27)
Definite, *n* (%)	22 (5)	408 (71)

Table 3 illustrates the diagnostic performance of the 3 versions of the Duke clinical criteria, considering episodes without IE (per reference standard) but classified as possible IE (per Duke clinical criteria) as true negatives. Sensitivity for the 2015 Duke-ESC, 2023 Duke-ESC, and 2023 Duke-ISCVID clinical criteria was calculated at 56% (95% CI: 52–60%), 61% (57–65%), and 71% (67–74%), respectively, while specificity stood at 100% (99–100%), 97% (95–98%), and 95% (93–97%), respectively. Table 4 illustrates the diagnostic performance of the 3 versions of the Duke clinical criteria, considering episodes without IE (per reference standard) but classified as possible IE (per Duke clinical criteria) as false positives. The specificity of the 2015 Duke-ESC, 2023 Duke-ESC, and 2023 Duke-ISCVID the clinical criteria was 67% (63–71%), 56% (51–61%), and 34% (29–38%), respectively.

**Table 3.** Performance of the Different Versions of the Duke Clinical Criteria Among Episodes With Suspected Infective Endocarditis and Intracardiac Prosthetic Material, Considering Patients Without IE (per Reference Standard) but Classified as Possible IE (per Duke Clinical Criteria) as True Negatives

	Sensitivity % (95% CI)	Specificity % (95% CI)	PPV % (95% CI)	NPV % (95% CI)	Accuracy % (95% CI)
All episodes with intracardiac prosthetic material (*n* = 1025)					
2015 Duke-ESC	56 (52–60)	100 (99–100)	100 (98–100)	64 (61–66)	75 (72–78)
2023 Duke-ESC	61 (57–65)	97 (95–98)	96 (93–97)	66 (63–68)	76 (74–79)
2023 Duke-ISCVID	71 (67–74)	95 (93–97)	95 (94–97)	71 (69–74)	81 (79–84)
Episodes with surgical prosthetic valve or transcatheter aortic valve replacement (*n* = 620)					
2015 Duke-ESC	60 (55–64)	100 (97–100)	100 (97–100)	57 (54–59)	73 (70–77)
2023 Duke-ESC	62 (57–67)	97 (94–99)	98 (95–99)	58 (54–61)	74 (71–78)
2023 Duke-ISCVID	73 (69–78)	95 (91–97)	96 (94–98)	65 (61–69)	81 (77–84)
Episodes with surgical prosthetic valve, or transcatheter aortic valve replacement and sufficient cardiac imaging exams (*n* = 502)					
2015 Duke-ESC	66 (61–71)	99 (96–100)	100 (97–100)	55 (51–58)	76 (72–79)
2023 Duke-ESC	67 (62–72)	98 (94–100)	99 (96–100)	55 (51–59)	76 (72–80)
2023 Duke-ISCVID	79 (75–84)	95 (90–98)	98 (95–99)	66 (61–70)	84 (81–87)
Episodes with transcatheter aortic valve replacement (*n* = 101)					
2015 Duke-ESC	41 (29–54)	100 (91–100)	100 (87–100)	49 (44–55)	62 (52–72)
2023 Duke-ESC	44 (31–57)	100 (91–100)	100 (88–100)	51 (45–56)	64 (54–74)
2023 Duke-ISCVID	63 (50–74)	100 (91–100)	100 (92–100)	61 (53–68)	76 (67–84)
Episodes with cardiac implantable electronic devices (*n* = 566)					
2015 Duke-ESC	53 (47–59)	100 (98–100)	99 (96–100)	67 (64–70)	76 (72–79)
2023 Duke-ESC	59 (53–65)	96 (93–98)	94 (90–97)	70 (67–73)	78 (74–81)
2023 Duke-ISCVID	66 (61–72)	95 (92–97)	93 (89–96)	73 (70–76)	80 (77–84)

Abbreviations: CI, confidence interval; ESC, European Society of Cardiology; ISCVID, International Society of Cardiovascular Infectious Disease; NPV, negative predictive value; PPV, positive predictive value.

**Table 4.** Performance of the Different Versions of the Duke Clinical Criteria Among Episodes With Suspected Infective Endocarditis and Intracardiac Prosthetic Material, Considering Patients Without IE (per Reference Standard) but Classified as Possible IE (per Duke Clinical Criteria) as False Positives

	Sensitivity % (95% CI)	Specificity % (95% CI)	PPV % (95% CI)	NPV % (95% CI)	Accuracy % (95% CI)
All episodes with intracardiac prosthetic material (*n* = 1025)					
2015 Duke-ESC	56 (52–60)	67 (63–71)	69 (65–72)	54 (51–57)	61 (58–64)
2023 Duke-ESC	61 (57–65)	56 (51–61)	64 (61–67)	53 (49–56)	59 (56–62)
2023 Duke-ISCVID	71 (67–74)	34 (29–38)	58 (56–60)	47 (43–52)	55 (51–58)
Episodes with surgical prosthetic valve or transcatheter aortic valve replacement (*n* = 620)					
2015 Duke-ESC	60 (55–64)	60 (54–67)	74 (70–77)	44 (40–48)	60 (56–64)
2023 Duke-ESC	62 (57–67)	60 (54–67)	75 (71–78)	46 (42–50)	62 (58–65)
2023 Duke-ISCVID	73 (69–78)	33 (27–40)	68 (65–70)	40 (34–46)	60 (56–63)
Episodes with surgical prosthetic valve, or transcatheter aortic valve replacement and sufficient cardiac imaging exams (*n* = 502)					
2015 Duke-ESC	66 (61–71)	66 (58–74)	82 (79–86)	45 (40–49)	66 (62–70)
2023 Duke-ESC	67 (62–72)	65 (57–73)	82 (79–85)	45 (40–50)	66 (62–70)
2023 Duke-ISCVID	79 (75–84)	34 (28–44)	75 (72–77)	42 (35–49)	67 (62–71)
Episodes with transcatheter aortic valve replacement (*n* = 101)					
2015 Duke-ESC	41 (29–54)	84 (68–94)	81 (66–91)	45 (39–51)	56 (46–66)
2023 Duke-ESC	44 (31–57)	65 (47–80)	68 (56–78)	40 (33–48)	51 (41–62)
2023 Duke-ISCVID	63 (50–74)	35 (20–53)	63 (55–69)	35 (24–48)	52 (42–63)
Episodes with cardiac implantable electronic devices (*n* = 566)					
2015 Duke-ESC	53 (47–59)	75 (69–80)	68 (63–73)	60 (57–64)	64 (59–68)
2023 Duke-ESC	59 (53–65)	57 (51–63)	59 (55–63)	57 (53–61)	58 (54–62)
2023 Duke-ISCVID	66 (61–72)	36 (30–42)	52 (49–55)	51 (45–56)	51 (47–56)

Abbreviations: CI, confidence interval; ESC, European Society of Cardiology; ISCVID, International Society of Cardiovascular Infectious Disease; NPV, negative predictive value; PPV, positive predictive value.

## DISCUSSION

Among the 3 versions of the Duke criteria, the ISCVID version demonstrated the highest sensitivity but at the cost of the lowest specificity.

Previous studies evaluating different iterations of the Duke criteria in various populations, such as patients with suspected IE or bacteremia, consistently showed that both 2023 versions improved sensitivity compared to the 2015 and 2000 versions [16, 18–23, 25–27]. In the present study, focusing on patients with prosthetic intracardiac material, the 2023 ESC version showed a modest increase in sensitivity (61%) compared to the 2015 ESC version (56%). However, the ISCVID version exhibited a more substantial improvement, reaching 71% sensitivity. The enhanced sensitivity of the 2023 ESC version can be partly attributed to the inclusion of [^18^F]FDG PET/CT positivity for CIED leads and the classification of CIED and TAVI as minor predisposing factors [15]. While these modifications were also incorporated into the ISCVID criteria, the superior sensitivity of the ISCVID version is likely driven by changes to the major microbiological criterion, particularly the inclusion of typical microorganisms in the setting of intracardiac prosthetic material [14]. As previously observed, the criteria perform better in patients with prosthetic valves than in those with CIEDs [18, 19], primarily due to the superior diagnostic performance of [^18^F]FDG PET/CT in the former group [8, 12]. Additionally, cardiac CT is a valuable tool for assessing prosthetic valves but has no diagnostic utility for suspected CIED-lead IE [9].

Data on patients with prosthetic valves remain limited [18, 19, 21, 25, 26]. A French study (*n* = 414) found no difference in sensitivity between the 2023 ISCVID and 2015 ESC criteria (97% for both), but specificity was lower with the 2023 ISCVID version (49% vs 57%) [19]. A Dutch study (*n* = 284) reported improved sensitivity for both 2023 versions (86% for ISCVID and 82% for ESC) compared to the 2015 ESC criteria (69%). Specificity remained high for the 2015 ESC and 2023 ISCVID versions (95%), while it decreased with the 2023 ESC criteria (82%) [18]. In a Swiss cohort, sensitivity was assessed in patients with confirmed PVE (*n* = 130), showing a greater increase with the 2023 ISCVID version (84%) compared to the 2023 ESC (73%) [21, 25].

A separate French study focusing on TAVI-related IE (*n* = 92) found a slight increase in sensitivity for both 2023 versions (ISCVID: 76%, ESC: 77%) compared to the 2015 ESC criteria (73%), while specificity remained consistent across all versions (90%) [26]. In the present study, the sensitivity of the different versions of the Duke criteria for patients with TAVI was lower than in the aforementioned study. This discrepancy can be explained by 2 key factors. First, our study included a higher proportion of TAVI patients in the suspected IE group whose diagnosis was ultimately rejected compared to the previous study (37% vs 11%). Second, a smaller percentage of patients in our study underwent [^18^F]FDG PET/CT and/or cardiac CT (42% vs 83%) [26].

Data on CIED-related IE are even scarcer, with only 2 studies evaluating different Duke criteria versions. In the French study (*n* = 284), the 2023 ISCVID version showed higher sensitivity (97%) compared to the 2015 ESC criteria (90%), but specificity decreased from 69% (2015 ESC) to 28% (2023 ISCVID) [19]. The Dutch study (*n* = 103) reported similar sensitivity for both 2023 versions (79%), which was higher than that of the 2015 ESC criteria (68%). Specificity remained high for the 2015 ESC and 2023 ISCVID versions (96%), while a decrease was observed with the 2023 ESC criteria (70%) [18].

All aforementioned studies have 3 main limitations [18, 19, 21, 25, 26], which the present study addresses. First, the sample sizes in prior studies were relatively small, limiting their ability to comprehensively assess diagnostic performance of complex criteria [18, 19, 21, 25, 26]. Our study is the largest to date, evaluating 1025 patients with prosthetic intracardiac material, including subsets of 537 patients with surgical prosthetic valve, 101 with TAVI, and 566 with CIED. Second, most previous studies predominantly included patients with IE, leading to an overestimation of sensitivity [18, 19, 21, 25, 26]. In contrast, our study included patients who underwent blood cultures and echocardiography for IE suspicion, the setting where diagnostic criteria are applied in clinical practice. Indeed, nearly half of the patients in this study had a clinical suspicion of IE that was ultimately rejected, distinguishing this work from previous studies that primarily focused on confirmed IE cases [18, 19, 21, 25, 26]. Third, prior studies classified patients without IE who fell into the possible IE category as true negatives, artificially inflating specificity [18, 19, 21, 25, 26]. We instead considered classification into possible IE a diagnostic failure, as these cases require further clinical assessment to confirm or exclude IE. To further address this issue, we conducted an additional analysis in which patients without confirmed IE (according to the reference standard) but classified as possible IE were categorized as false positives. This approach resulted in a substantial drop in specificity across all versions (previously ≥95% for all versions of the Duke criteria). With the new analysis, 2023 ISCVID specificity was the lowest (34%) compared to 2023 ESC (56%) and 2015 ESC (67%). The decreased specificity of the 2023 ISCVID version can be attributed to the same factors responsible for its increased sensitivity, particularly the expansion of the typical microorganism category [14]. Notably, 18% of episodes without IE involved positive blood cultures with a newly classified typical microorganism in the presence of intracardiac prosthetic material. As previously demonstrated, the inclusion of coagulase-negative staphylococci and *Candida* spp. in this group significantly enhanced the diagnostic performance of the 2023 ISCVID criteria, whereas the addition of *Pseudomonas aeruginosa* and *Serratia marcescens* had the opposite effect [23].

The Duke criteria were originally developed to standardize IE research by maximizing sensitivity and PPV, ensuring that cases classified as definite IE almost always reflect true infections [14, 15, 28]. Subsequent evaluations have demonstrated improved sensitivity with both of the 2023 Duke revisions [16–23]. However, in clinical practice, diagnostic tools must also prioritize high specificity and NPV to confidently rule out disease in patients who do not meet the diagnostic thresholds. In clinical practice, this trade-off is critical. A diagnostic rule that lacks specificity may lead to overdiagnosis and unnecessary treatment, particularly in patients with comorbidities or indwelling prosthetic material who may fulfill major or minor Duke criteria without having true IE. This can result in prolonged antibiotic courses, hospital stays, and even unwarranted surgical interventions—each carrying significant risks. Additionally, the “possible IE” category, by design, introduces diagnostic ambiguity and limits the criteria's utility for guiding real-time clinical decision-making. In the context of research, the lower specificity means that studies relying solely on the Duke classification may overestimate the number of true IE cases, especially when including “possible IE” without further clinical adjudication. This can affect the accuracy of outcome comparisons, risk prediction models, and evaluations of diagnostic tools or therapeutic interventions.

Another notable finding was that the majority of episodes involving typical IE pathogens in patients with prosthetic intracardiac material, such as *Staphylococcus aureus*, streptococci, enterococci, and coagulase-negative staphylococci, were both diagnosed and treated as IE [10, 11, 29]. In particular, two-thirds of *S. aureus* bacteremia episodes led to an IE diagnosis. In some instances, patients were treated for IE even without undergoing the full range of cardiac imaging modalities. TEE, [¹⁸F]FDG PET/CT, or cardiac CT were not performed in all cases, often due to patient refusal, advanced age, or significant comorbidities. Nonetheless, in the absence of a clear alternative diagnosis, treatment decisions were based on clinical suspicion. These findings underscore the importance of maintaining a high index of suspicion for IE in patients with prosthetic intracardiac devices and bacteremia with typical pathogens, and they highlight the need for a comprehensive diagnostic workup whenever feasible.

The present study has several limitations. First, it was conducted at 2 Swiss tertiary institutions, where infectious disease specialists systematically evaluate all suspected IE cases and advanced imaging modalities, such as [^18^F]FDG PET/CT, cardiac CT for valvular and paravalvular assessment, and cerebral and thoracoabdominal imaging for embolic event detection, are readily available. As a result, our findings may have limited generalizability to other healthcare settings. Despite the broad availability of [^18^F]FDG PET/CT and cardiac CT, not all patients with a surgical prosthetic valve or transcatheter aortic valve replacement who were considered at high risk of IE by the consulting infectious diseases specialist underwent these imaging examinations. The main reasons were advanced age, substantial comorbidities, or patient refusal, reflecting real-life clinical practice. Moreover, the use of [^18^F]FDG PET/CT increased over time, particularly after the of a multidisciplinary Endocarditis Team [30]. To account for these potential biases, we performed an additional analysis focusing on patients with surgical prosthetic valves or TAVR who had undergone what we defined as sufficient cardiac imaging (Tables 3 and 4), allowing for a more consistent evaluation of the Duke criteria's diagnostic performance in this subgroup. Compared to the overall cohort, we observed a slight increase in both sensitivity and specificity among patients with sufficient imaging. Additionally, the use of both Endocarditis Teams (in prospective cohorts) and expert clinicians (in retrospective cohorts) to adjudicate cases may have introduced some degree of misclassification. However, this approach was necessary given the absence of a definitive gold standard for diagnosing IE, which requires a multidisciplinary expert evaluation to ensure diagnostic accuracy and guide optimal management. This reflects the necessity of a detailed, expert-driven strategy to achieve accurate diagnosis and effective management of IE. To mitigate this potential classification bias, we have transparently reported all cases where the expert adjudicated diagnosis differed from the classification based on the Duke criteria (Supplementary Tables 2 and 3).

In conclusion, the 2023 ISCVID clinical criteria demonstrated the highest sensitivity for diagnosing IE in the presence of intracardiac prosthetic material compared to the 2015 and 2023 ESC criteria. However, this increased sensitivity came at the expense of specificity, which was lower with the 2023 ISCVID criteria. Notably, the improved sensitivity and reduced specificity were largely attributable to the inclusion of newly classified typical microorganisms in the presence of prosthetic intracardiac material. Future studies should assess the impact of these microbiological modifications proposed by ISCVID.

## Supplementary Material

ofaf507_Supplementary_Data

## Supplementary Data

Supplementary materials are available at *Open Forum Infectious Diseases* online. Consisting of data provided by the authors to benefit the reader, the posted materials are not copyedited and are the sole responsibility of the authors, so questions or comments should be addressed to the corresponding author.
