# Supplementary material for: Performance of Different Versions of Duke Criteria in Diagnosing Infective Endocarditis in Patients With Intracardiac Prosthetic Materials
Source: Open Forum Infect Dis. 2025 Aug 19;12(9):ofaf507. doi: 10.1093/ofid/ofaf507 (PMC12404897; doi:10.1093/ofid/ofaf507)
Supplement: ofaf507_Supplementary_Data [file ofaf507_supplementary_data.docx]

**Supplementary Table 1.** Performance of cardiac imaging and site of infective endocarditis based on the type of prosthetic intracardiac material

|  | **SVR (n=537)** | **TAVI (n=101)** | **CIED (n=566)** | **LVAD (n=45)** |
| --- | --- | --- | --- | --- |
| Cardiac imaging |  |  |  |  |
| TTE, n (%) | 461 (86) | 91 (90) | 466 (82) | 29 (64) |
| TEE, n (%) | 319 (59) | 47 (47) | 288 (51) | 20 (44) |
| [¹⁸F]FDG PET/CT, n (%) | 164 (31) | 38 (38) | 178 (31) | 19 (42) |
| Cardiac CT, n (%) | 44 (8) | 7 (7) | 19 (3) | 1 (2) |
| Concomitant prosthetic intracardiac material |  |  |  |  |
| SVR, n (%) | - | 18 (18) | 145 (26) | 4 (9) |
| TAVI, n (%) | 18 (3) | - | 46 (8) | 0 (0) |
| CIED, n (%) | 145 (27) | 46 (46) | - | 26 (58) |
| LVAD, n (%) | 4 (0.7) | 0 (0) | 26 (5) | - |
| Infective endocarditis | 357 (67) | 64 (64) | 288 (51) | 14 (31) |
| Prosthetic valve (SVR or TAVI), n (%) | 312 (87) | 54 (84) | 89 (31) | 0 (0) |
| CIED-lead, n (%) | 41 (11) | 13 (20) | 191 (66) | 12 (86) |
| LVAD-cannula, n (%) | 0 (0) | 0 (0) | 0 (0) | 0 (0) |
| Native valve, n (%) | 41 (11) | 10 (16) | 79 (27) | 2 (14) |

[^18^F]FDG PET/CT: [^18^F]Fluorodeoxyglucose Positron Emission Tomography/Computed Tomography; CIED: cardiac implantable electronic device; LVAD: Left ventricular assist device; SVR: surgical prosthetic valve; TAVI: transcatheter aortic valve implantation; TEE: transesophageal echocardiograph; TTE: transthoracic echocardiograph

**Supplementary Table 2.** Episodes of infective endocarditis (reference standard) classified as rejected infective endocarditis by any version of the Duke clinical criteria

|  | **Microbiologic criterion (positive bcs)** | **Imaging criterion** | **Predisposition** | **Fever** | **Vascular or immunologic phenomena** | **Other information** |
| --- | --- | --- | --- | --- | --- | --- |
| 1 | *S. epidermidis* (2 bcs) | CIED-lead vegetation | CIED | N |  | CIED-extraction; CIED-lead culture positive for *S. epidermidis* with pocket culture negative |
| 2 | *S. hominis* (2 bcs) | Valve small mobile element | CIED | N | Janeway lesions | CIED-extraction; CIED-lead culture positive for *S. hominis* with pocket culture negative |
| 3 | *S. epidermidis* (2 bcs) | Significant new valvular regurgitation | TAVI | Y |  | Bacteremia of unknown origin; [^18^F]FDG PET/CT not performed |
| 4 | *E. feacium* (2 bcs) | Significant new valvular regurgitation | TAVI, CIED | Y |  | Bacteremia of unknown origin; [^18^F]FDG PET/CT performed >7 days from bacteremia onset (negative result) |
| 5 | *E. faecalis* (2 bcs) | Valve leaflet thickening | TAVI, CIED | Y |  | Nosocomial bacteremia of unknown origin |
| 6 | *E. faecalis* (1 bcs) |  | Prosthetic valve, CIED | Y |  | Bacteremia of unknown origin; [^18^F]FDG PET/CT not performed |
| 7 | *E. faecalis* (2 bcs) | Valve small mobile element | CIED | Y |  | Nosocomial bacteremia of unknown origin; patient’s refusal of TOE |
| 8 |  | Valve perforation; CIED-lead small element | CIED | N |  | Antibiotic treatment before blood culture collection; CIED extraction; CIED-lead culture positive for *P. aeruginosa* with pocket culture negative |
| 9 | *K. pneumoniae* (2 bcs) | CIED-lead vegetation | CIED | N |  | CIED extraction; CIED-lead culture positive for *K. pneumoniae* with pocket culture negative |
| 10 |  | CIED-lead vegetation | CIED | N |  | Antibiotic treatment before blood culture collection; CIED extraction; CIED-lead culture positive for *S. epidermidis* with pocket culture negative |
| 11 |  | CIED-lead vegetation | CIED | N |  | Sepsis; antibiotic treatment before blood culture collection; CIED extraction |
| 12 | *E. faecium* (2 bcs) | Abnormal metabolic activity ([^18^F]FDG PET/CT) of CIED-lead | CIED | N |  |  |
| 13 | *E. coli* (2 bcs) | CIED-lead small element | CIED | Y |  | Sepsis; bacteremia of unknown origin; CIED extraction |
| 14 |  | Valve leaflet thickening | Prosthetic valve, CIED | Y |  | Antibiotic treatment before blood culture collection; valve surgery; macroscopic signs of IE; positive pathology for acute IE |
| 15 | *S. lugdunensis* (2 bcs) | CIED-lead small element | CIED | Y |  | Spondylodiscitis; CIED extraction |
| 16 |  | CIED-lead vegetation | CIED | N |  | Antibiotic treatment before blood culture collection; CIED extraction; CIED-lead culture positive for *E. cloacae* with pocket culture negative |
| 17 |  | CIED-lead vegetation | CIED | N |  | Antibiotic treatment before blood culture collection; CIED extraction; CIED-lead culture positive for *S. epidermidis* with pocket culture negative |
| 18 |  | CIED-lead vegetation | CIED | N |  | Antibiotic treatment before blood culture collection; CIED extraction |
| 19 |  |  | CIED | Y | Cerebral emboli | Antibiotic treatment before blood culture collection; CIED extraction; CIED-lead culture positive for *S. marcescens* with pocket culture negative |
| 20 | *S. epidermidis* (2 bcs) | Valve leaflet thickening | Prosthetic valve | Y |  |  |
| 21 | *S. aureus* (2 bcs) | CIED-lead small element | CIED | N |  | CIED extraction; CIED-lead culture positive for *E. cloacae* with pocket culture negative |
| 22 | *E. faecalis* (2 bcs) |  | CIED | N |  | Bacteremia of unknown origin |
| 23 | *S. epidermidis* (2 bcs) | CIED-lead small element | CIED | N |  | CIED extraction; CIED-lead culture positive for *E. cloacae* with pocket culture negative |
| 24 |  | CIED-lead small element | CIED | N |  | Antibiotic treatment before blood culture collection; CIED extraction; CIED-lead culture positive for *S. aureus* with pocket culture negative |
| 25 | *C. albicans* (2 bcs) | CIED-lead small element | CIED | N |  | Candidemia of unknown origin; CIED extraction |
| 26 | *P. aeruginosa* (2 bcs) |  | IVDU, CIED | N |  | Persistent bacteremia of unknown origin |
| 27 |  | Significant new valvular regurgitation | Prosthetic valve, CIED | N |  | Antibiotic treatment before blood culture collection |
| 28 | *S. marcescens* (1 bcs) | Valve small mobile element | Prior IE, prosthetic valve | Y |  | Bacteremia of unknown origin |
| 29 |  | Abscess | TAVI | N |  | Antibiotic treatment before blood culture collection; valve surgery; macroscopic signs of IE |
| 30 | *S. epidermidis* (2 bcs) |  | CIED | Y | Roth spots | CIED extraction; CIED-lead culture positive for *S. epidermidis* with pocket culture negative |
| 31 |  | Significant new valvular regurgitation | Prior IE, prosthetic valve | N |  | Antibiotic treatment before blood culture collection; valve surgery; macroscopic signs of IE; positive valve culture for *S. epidermidis* |
| 32 | *S. epidermidis* (2 bcs) |  | Prior IE, prosthetic valve, CIED | N |  | Valve surgery; macroscopic signs of IE; positive valve culture for *S. epidermidis;* CIED extraction; CIED-lead culture positive for *S. epidermidis* with pocket culture negative |
| 33 | *S. epidermidis* (2 bcs) |  | Prosthetic valve | Y |  | Persistent bacteremia of unknown origin |
| 34 | *S. epidermidis* (2 bcs) | Valve small mobile element | Prosthetic valve, CIED | Y |  | Bacteremia of unknown origin |
| 35 | *S. epidermidis* (2 bcs) |  | CIED | N |  | CIED extraction; CIED-lead culture positive for *S. epidermidis* with pocket culture negative |
| 36 | *S. aureus* (1 bcs) |  | CIED | Y |  | Antibiotic treatment before blood culture collection; bacteremia of unknown origin |
| 37 | *S. gallolyticus* (2 bcs) |  | Prior IE, prosthetic valve, CIED | N |  | Bacteremia of unknown origin; patient’s refusal of TEE and [^18^F]FDG PET/CT; |
| 38 |  | Valve leaflet thickening | Prosthetic valve | Y |  | Antibiotic treatment before blood culture collection |
| 39 | *S. aureus* (1 bcs) |  | CIED | N |  | Antibiotic treatment before blood culture collection; bacteremia of unknown origin; patient’s refusal of TEE and [^18^F]FDG PET/CT |
| 40 |  | CIED-lead small element | Prior IE, CIED | N |  | Antibiotic treatment before blood culture collection; CIED extraction; CIED-cable culture positive for *S. aureus* with pocket culture negative |
| 41 |  | CIED-lead small element | CIED | N |  | Antibiotic treatment before blood culture collection; CIED extraction; CIED-cable culture positive for *S. epidermidis* with pocket culture negative |
| 42 |  | CIED-lead small element | CIED | N |  | Antibiotic treatment before blood culture collection; CIED extraction; CIED-cable culture positive for *S. epidermidis* with pocket culture negative |
| 43 |  | CIED-lead small element | CIED | N |  | Antibiotic treatment before blood culture collection; CIED extraction; CIED-cable culture positive for *C. acnes* with pocket culture negative |
| 44 |  | CIED-lead small element | Prosthetic valve, CIED | N |  | Antibiotic treatment before blood culture collection; CIED extraction; CIED-cable culture positive for *S. epidermidis* with pocket culture negative |
| 45 |  | CIED-lead small element | CIED | N |  | Antibiotic treatment before blood culture collection; CIED extraction; CIED-cable culture positive for *S. aureus* with pocket culture negative |
| 46 | *P. micra* (2 bcs) | CIED-lead small element | CIED | N |  | Bacteremia of unknown origin |
| 47 | *S. aureus* (1 bcs) | CIED-lead small element | Prosthetic valve, CIED | N |  | Antibiotic treatment before blood culture collection; CIED extraction; CIED-cable culture positive for *S. aureus* with pocket culture negative |

[^18^F]FDG PET/CT: [^18^F]Fluorodeoxyglucose Positron Emission Tomography/Computed Tomography; bcs: blood culture set; CIED: cardiac implantable electronic device; IE: infective endocarditis; IVDU: intravenous drug use; N: no; TAVI: transcatheter aortic valve implantation; TEE: transesophageal echocardiography; Y: yes

**Supplementary Table 3.** Episodes without infective endocarditis (reference standard) classified as definite infective endocarditis by any version of the Duke clinical criteria

|  | **Microbiologic criterion (positive bcs)** | **Imaging criterion** | **Predisposition** | **Fever** | **Vascular or immunologic phenomena** | **Other information** |
| --- | --- | --- | --- | --- | --- | --- |
| 1 |  | Valve vegetation | Prosthetic valve; CIED | Y | Cerebral emboli | Marantic endocarditis; valve operation at day 1; negative pathology and culture; antibiotic treatment for 7 days; no episode of bacteremia/IE in the subsequent 120 days |
| 2 | *S. aureus* (2 bcs),  *S. anginosus* (3 bcs) |  | IVDU; prosthetic valve | Y | Pulmonary emboli | Septic deep vein thrombosis related to IVDU; TEE, [^18^F]FDG PET/CT negative for IE |
| 3 | *E. faecalis* (2 bcs) | Valve leaflet thickening | Prosthetic valve | Y |  | Urinary-tract infection; TEE negative for IE; no recurrence of bacteremia in the subsequent 120 days |
| 4 |  | Valve small mobile element; significant new valvular regurgitation | Prosthetic valve; CIED | Y | Cerebral emboli | Marantic endocarditis; valve operation at day 7; negative pathology and culture; no antibiotic treatment before or after operation; no episode of bacteremia/IE in the subsequent 120 days |
| 5 | *S. aureus* (2 bcs) |  | CIED | Y | Pulmonary emboli | Septic deep vein thrombosis related to IVDU; TEE and [^18^F]FDG PET/CT negative for IE |
| 6 | *S. aureus* (2 bcs) |  | Prosthetic valve | Y | Janeway lesions | Urinary-tract infection; TTE and ^18^F-FDG PET/CT negative for IE; petechia due to septic shock resembling Janeway lesions; no recurrence of bacteremia in the subsequent 120 days |
| 7 | *S. aureus* (1 bcs) |  | CIED | Y | Pulmonary emboli | Persistent bacteremia; catheter-related infection with septic deep vein thrombosis; TEE and [^18^F]FDG PET/CT negative for IE; no recurrence of bacteremia in the subsequent 120 days |
| 8 | *S. aureus* (2 bcs) | Valve small mobile element; significant new valvular regurgitation | CIED | N |  | Catheter-related infection; TEE and [^18^F]FDG PET/CT negative for IE; no recurrence of bacteremia in the subsequent 55 days |
| 9 | *C. albicans* (2 bcs) |  | Prosthetic valve | Y | Pulmonary emboli | Intraabdominal infection with septic deep vein thrombosis; TEE negative for IE; no recurrence of candidemia in subsequent 61 days |
| 10 | *S. aureus* (2 bcs) | Significant new valvular regurgitation | Prosthetic valve | Y |  | Catheter-related infection with septic deep vein thrombosis; TEE and [^18^F]FDG PET/CT negative for IE; no recurrence of bacteremia in the subsequent 120 days |
| 11 | *S. aureus* (2 bcs) | Significant new valvular regurgitation | Prosthetic valve | Y |  | Skin and soft tissue infection; TEE and [^18^F]FDG PET/CT negative for IE; no recurrence of bacteremia in the subsequent 120 days |
| 12 | *E. faecium* (3 bcs) |  | CIED | Y | Pulmonary emboli | Catheter-related infection with septic deep vein thrombosis; TEE and [^18^F]FDG PET/CT negative for IE; no recurrence of bacteremia in the subsequent 29 days |
| 13 | *C. hominis* (1 bcs) | Significant new valvular regurgitation | Prosthetic valve | Y |  | Dental abscess; TEE and [^18^F]FDG PET/CT negative for IE; no recurrence of bacteremia in the subsequent 120 days |
| 14 | *L. garviae* (4 bcs),  *E. coli* (1 bcs) |  | Prosthetic valve | Y | Cerebral emboli | Spondylodiscitis; TEE and [^18^F]FDG PET/CT negative for IE; ischemic stroke at day 9 due to atrial fibrillation; no recurrence of bacteremia in the subsequent 120 days |
| 15 | *S. anginosus* (2 bcs) | Significant new valvular regurgitation | CIED | Y |  | Bacteremia of unknown origin; TEE negative for IE; no recurrence of bacteremia in the subsequent 120 days |
| 16 | *S. aureus* (2 bcs) | Significant new valvular regurgitation | Prosthetic valve; CIED | Y |  | Catheter-related infection; TEE and [^18^F]FDG PET/CT negative for IE; no recurrence of bacteremia in the subsequent 46 days |
| 17 | *S. anginosus* (3 bcs) | Significant new valvular regurgitation | Prosthetic valve | Y |  | Cholangitis; TEE negative for IE; no recurrence of bacteremia in the subsequent 12 days |
| 18 | *S. mitis* (2 bcs) | Significant new valvular regurgitation | CIED | Y |  | CIED-pocket infection; TEE and [^18^F]FDG PET/CT negative for IE; CIED extraction; CIED-lead culture negative; no recurrence of bacteremia in the subsequent 120 days |
| 19 | *S. marcescens* (2 bcs) |  | Prosthetic valve | Y | Cerebral emboli | Catheter-related infection; TEE negative for IE; ischemic stroke at day 7 due to atrial fibrillation; no recurrence of bacteremia in the subsequent 9 days |
| 20 | *E. faecalis* (2 bcs) | Significant new valvular regurgitation | CIED | Y |  | Bacteremia of unknown origin; TEE and [^18^F]FDG PET/CT negative for IE; no recurrence of bacteremia in the subsequent 120 days |
| 21 | *E. faecalis* (2 bcs) | Significant new valvular regurgitation | CIED | N |  | Bacteremia of unknown origin; TEE and [^18^F]FDG PET/CT negative for IE; no recurrence of bacteremia in the subsequent 22 days |
| 22 | *P. aeruginosa* (3 bcs) |  | CIED | Y | Pulmonary emboli | Catheter-related infection with septic deep vein thrombosis; TEE and [^18^F]FDG PET/CT negative for IE; no recurrence of bacteremia in the subsequent 120 days |
| 23 | *S. epidermidis* (2 bcs) | Significant new valvular regurgitation | CIED | Y |  | Catheter-related infection; TEE and [^18^F]FDG PET/CT negative for IE; no recurrence of bacteremia in the subsequent 99 days |
| 24 | *S. aureus* (2 bcs) | Valve leaflet thickening | CIED | N |  | Skin and soft tissue infection; TEE and [^18^F]FDG PET/CT negative for IE; no recurrence of bacteremia in the subsequent 35 days |
| 25 | *C. albicans* (2 bcs) | Significant new valvular regurgitation | CIED | Y |  | Catheter-related infection; TEE and [^18^F]FDG PET/CT negative for IE; no recurrence of bacteremia in the subsequent 120 days |
| 26 | *S. aureus* (2 bcs) |  | CIED | Y |  | Spondylodiscitis; TEE negative for IE; no recurrence of bacteremia in the subsequent 38 days |
| 27 | *S. aureus* (2 bcs) |  | CIED | Y |  | Spondylodiscitis; TEE and [^18^F]FDG PET/CT negative for IE; no recurrence of bacteremia in the subsequent 53 days |
| 28 | *S. aureus* (2 bcs),  *K. pneumoniae* (2 bcs) | Valve leaflet thickening | CIED | Y |  | Urinary-tract infection related to urinary catheter; TEE negative for IE; no recurrence of bacteremia in the subsequent 120 days |
| 29 | *S. aureus* (2 bcs) | Valve leaflet thickening | Prosthetic valve | Y |  | Prosthetic joint infection; TEE negative for IE; no recurrence of bacteremia in the subsequent 120 days |
| 30 | *S. aureus* (1 bcs) |  | CIED | Y |  | Spondylodiscitis; persistent bacteremia; TEE and [^18^F]FDG PET/CT negative for IE; no recurrence of bacteremia in the subsequent 120 days |
| 31 | *S. aureus* (2 bcs) |  | CIED | Y | Splenic emboli | CIED-pocket infection; TEE and [^18^F]FDG PET/CT negative for IE; CIED extraction; CIED-lead culture negative; no recurrence of bacteremia in the subsequent 120 days |
| 32 | *E. faecalis* (2 bcs) |  | Prosthetic valve | Y |  | Spondylodiscitis; TEE negative for IE; no recurrence of bacteremia in the subsequent 120 days |

[^18^F]FDG PET/CT: [^18^F]Fluorodeoxyglucose Positron Emission Tomography/Computed Tomography; bcs: blood culture set; CIED: cardiac implantable electronic device; IE: infective endocarditis; N: no; TEE: transesophageal echocardiography; Y: yes
